# Chest wall desmoid tumours treated with definitive radiotherapy: a plan comparison of 3D conformal radiotherapy, intensity-modulated radiotherapy and volumetric-modulated arc radiotherapy

**DOI:** 10.1186/s13014-016-0611-0

**Published:** 2016-03-02

**Authors:** Jia Liu, Diana Ng, James Lee, Paul Stalley, Angela Hong

**Affiliations:** Central Clinical School, Faculty of Medicine, University of Sydney, Camperdown, NSW Australia; Department of Radiation Oncology, Mater Hospital, Genesis Cancer Care, St Leonards, NSW Australia; Bone and Soft Tissue Sarcoma Unit, Royal Prince Alfred Hospital, Camperdown, NSW Australia

## Abstract

**Purpose:**

Definitive radiotherapy is often used for chest wall desmoid tumours due to size or anatomical location. The delivery of radiotherapy is challenging due to the large size and constraints of normal surrounding structures. We compared the dosimetry of 3D conformal radiotherapy (3DCRT), intensity-modulated radiotherapy (IMRT) and volumetric-modulated arc radiotherapy (VMAT) to evaluate the best treatment option.

**Methods and materials:**

Ten consecutive patients with inoperable chest wall desmoid tumours (PTV range 416–4549 cm^3^) were selected. For each patient, 3DCRT, IMRT and VMAT plans were generated and the Conformity Index (CI), organ at risk (OAR) doses and monitor unit (MU) were evaluated. The Wilcoxon signed-rank test was used to compare dose delivered to both target and OARs.

**Results:**

The mean number of fields for 3DCRT and IMRT were 6.3 ± 2.1, 7.2 ± 1.8. The mean number of arcs for VMAT was 3.7 ± 1.1. The mean conformity index of VMAT (0.98 ± 0.14) was similar to that of IMRT (1.03 ± 0.13), both of which were significantly better than 3DCRT (1.35 ± 0.20; *p* = 0.005).

The mean dose to lung was significantly higher for 3DCRT (11.9Gy ± 7.9) compared to IMRT (9.4Gy ± 5.4, *p* = 0.014) and VMAT (8.9Gy ± 4.5, *p* = 0.017). For the 3 females, the low dose regions in the ipsilateral breast for VMAT were generally less with VMAT. IMRT plans required 1427 ± 532 MU per fraction which was almost 4-fold higher than 3DCRT (313 ± 112, *P* = 0.005). Compared to IMRT, VMAT plans required 60 % less MU (570 ± 285, *P* = 0.005).

**Conclusions:**

For inoperable chest wall desmoid tumours, VMAT delivered equivalent target coverage when compared to IMRT but required 60 % less MU. Both VMAT and IMRT were superior to 3DCRT in terms of better PTV coverage and sparing of lung tissue.

## Introduction

Desmoid-type fibromatosis is a locally aggressive, non-metastasizing mesenchymal tumour characterised by clonal proliferation of myofibroblasts [1]. Although rare, it is a highly heterogenous disease that has unpredictable behaviour and can arise almost anywhere in the body [2]. Subtypes based upon anatomical location include intra-abdominal fibromatosis, abdominal wall fibromatosis, and extra-abdominal fibromatosis [3]. Extra-abdominal fibromatoses mainly affect individuals between puberty and forty years of age and arise in a variety of anatomic locations, including the shoulder, chest wall, back and thigh [1]. Traditionally, wide surgical excision was considered to be the standard of care. However, due to their pattern of infiltrative and nonencapsulated growth, surgery can be associated with considerable functional and cosmetic morbidity, and high local recurrences rates [4–6]. Consequently, an upfront wait and see approach has been favoured in recent years, with treatment mandated in the case of disease progression [2].

Definitive radiotherapy (RT) plays an important role in the treatment of desmoid tumours that are inoperable due to size or anatomical location. In a comparative retrospective review, definitive RT in a total of 102 patients treated with a dose between 50 and 60 Gy resulted in local control rates of 78 % (80/102), significantly higher than that for surgery alone (61 %; *P* = 0.022) and no different when compared with surgery and adjuvant RT (75 %) [7]. However, the delivery of RT can be challenging due to the large size of some desmoid tumours and constraints of normal surrounding tissues. For example, chest wall desmoid tumours lie close to key organs including heart, lung and brachial plexus. As published RT series for the treatment of desmoid tumours have small patient numbers often spread over many years there has been wide variation in the reported RT dose, fractionation and technique, making it difficult to ascertain evidence-based data on optimal RT techniques. Furthermore, no study has previously compared RT planning techniques to optimize dose to inoperable desmoid tumours while minimising dose to major adjacent organs at risk.

We compared the performance of 3D conformal radiotherapy (3DCRT), intensity-modulated radiotherapy (IMRT) and volumetric-modulated arc radiotherapy (VMAT) to evaluate the best treatment technique for inoperable large volume chest wall desmoid tumour.

## Materials and methods

The study group comprised of 10 consecutive patients with inoperable chest wall desmoid tumours diagnosed between 2009 and 2013. All patients were assessed in a multidisciplinary clinic prior to undergoing RT. The study was approved by the ethics committee. For each patient, optimised 3DCRT, IMRT and VMAT plans were generated to a prescribed dose of 56Gy in 28 fractions based upon previously reported recommendations [8, 9]. The first three patients (patients 1–3) were planned using 3DCRT, IMRT and VMAT, prior to the decision to proceed with VMAT for treatment. The remainder of the patients had VMAT plans generated for their treatment with 3D and IMRT plans generated retrospectively for the purpose of this study.

The Gross Tumour Volume (GTV) was defined based upon each patient’s MRI. The Clinical Target Volume (CTV) was defined by expanding the GTV by 4–5 cm unless the area was bound by another anatomical structure. The Planning Target Volume (PTV) was defined as the CTV + 0.5 cm. An online strategy was employed for acquisition of images and their verification and correction prior to treatment. Organs at risk (OAR) considered in the treatment plan included heart, lung and breasts (in female patients).

A comparison of the Homogeneity Index (defined as difference between the dose covering 5 % and dose covering 95 % of the PTV), Conformity Index (defined as the ratio between the volume receiving at least 95 % of the prescribed dose and the PTV), OAR doses, monitor units (MUs) and treatment delivery time was evaluated. The Wilcoxon signed-rank test was used to compare dose delivered to both target and OARs. Statistical analyses were performed in SPSS (Version 22).

## Results

Table 1 summarises the baseline demographics of the ten patients in the study. The age range was 20 to 64. Three subjects were female and seven were male. Three tumours were right sided and seven were left sided. All tumours were large; a feature which made surgery a less desirable treatment modality, with the PTV volume ranging from 416 to 4539 cm^3^.

**Table 1:** Baseline demographics of subjects

Patient Number	Age	Gender	Tumour location	Initial MRI Dimensions (cm)	PTV volume (cm^3^)
1	29	F	L anterior chest wall	6.5 × 3.6 × 2.1	534
2	47	M	R posterior chest wall	10.0 × 3.5 × 5.5	2082
3	64	M	R anterior chest wall	6.9 × 2.6 × 3.0	767
4	34	F	L axilla	7.6 × 11.8 × 11.5	1017
5	34	M	L axilla and lateral chest wall	10.5 × 12.5 × 5.0	3896
6	22	M	R axilla and supraclavicular fossa	10.3 × 5.9 × 5.2	1303
7	46	M	R anteriolateral chest wall	15.0 × 20.0 × 6.0	4539
8	49	M	L anterior chest wall	8.5 × 5.6 × 6.8	950
9	41	F	L anterior chest wall	4.3 × 1.4 × 2.5	416
10	20	M	L neck and supraclavicular fossa	8.7 × 5.5 × 6.7	646

The mean number of fields (± standard deviation) for 3DCRT and IMRT were 6.3 ± 2.1 and 7.2 ± 1.8 respectively. The mean number of arcs for VMAT was 3.7 ± 1.1. Table 2 summarises the dosimetric results for the PTV. The minimum dose to the PTV was significantly higher for 3DCRT compared to IMRT and VMAT plans, while there was no difference in the maximum or mean dose to PTV. Due to significant inter-individual variability in the homogeneity index within each plan type (particularly for VMAT), the overall homogeneity index was statistically similar for the three plan types. The mean conformity index of VMAT (0.98 ± 0.14) was similar to that of IMRT (1.03 ± 0.13), both of which were significantly better than 3DCRT (1.35 ± 0.20; *p* = 0.005).

**Table 2:** Summary of dosimetric results for PTV

Parameter	3DCRT	IMRT	VMAT	*P* value		
				3DCRT vs IMRT	3DCRT vs VMAT	IMRT vs VMAT
Minimum (Gy)	40.0 ± 8.8	32.5 ± 11.2	30.57 ± 13.5	0.028	0.037	0.508
Maximum (Gy)	63.8 ± 3.0	67.5 ± 8.9	63.9 ± 2.9	0.203	0.959	0.333
Mean (Gy)	58.2 ± 1.9	57.5 ± 2.0	57.1 ± 1.4	0.092	0.074	0.878
D_5%_(Gy)	61.6 ± 2.2	60.5 ± 3.0	60.3 ± 2.5	0.169	0.037	0.859
D_95%_(Gy)	55.3 ± 2.8	53.3 ± 2.8	50.0 ± 8.6	0.203	0.053	0.114
HI (D5 % -D95 % ; Gy)	6.27 ± 2.76	7.20 ± 4.60	10.25 ± 10.42	0.575	0.386	0.139
V95 % (%)	95.5 ± 3.2	92.2 ± 7.2	92.5 ± 5.6	0.333	0.139	0.799
CI	1.35 ± 0.2	1.03 ± 0.13	0.98 ± 0.14	0.005	0.005	0.445
MU	313 ± 112	1427 ± 532	570 ± 285	0.005	0.432	0.005
Beam on time (second)	206 ± 122	302 ± 86	366 ± 164	0.056	0.005	0.231

*CI* Conformity index, defined as the ratio between the volume receiving at least 95 % of the prescribed dose and the volume of the PTV, *HI* Homogeneity index, defined as difference between the dose covering 5 % and dose covering 95 % of the PTV. *P* value determined using Wilcoxon matched pair rank sum test in SPSS

IMRT plans required 1427 ± 532 MU per fraction which was almost 4-fold higher than 3DCRT (313 ± 112, *P* = 0.005). Compared to IMRT, VMAT plans required 60 % less MU on the machine (570 ± 285, *P* = 0.005). The mean beam on time was 206 ± 122 s for 3DCRT, which was significantly lower than that of VMAT (366 ± 164 s, *P* = 0.005) and marginally lower than that of IMRT (302 ± 86 s, *P* = 0.056).

**Fig. 1:**
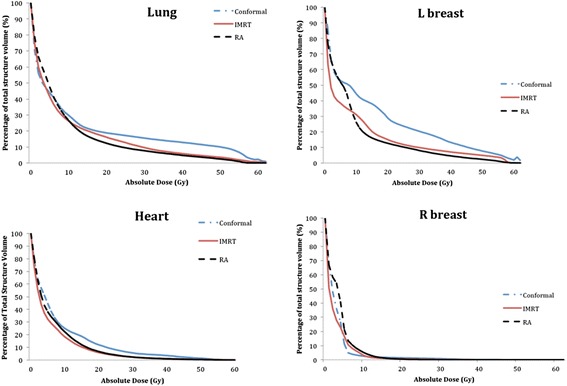
Mean Dose Volume Histograms for OAR

**Fig. 2:**
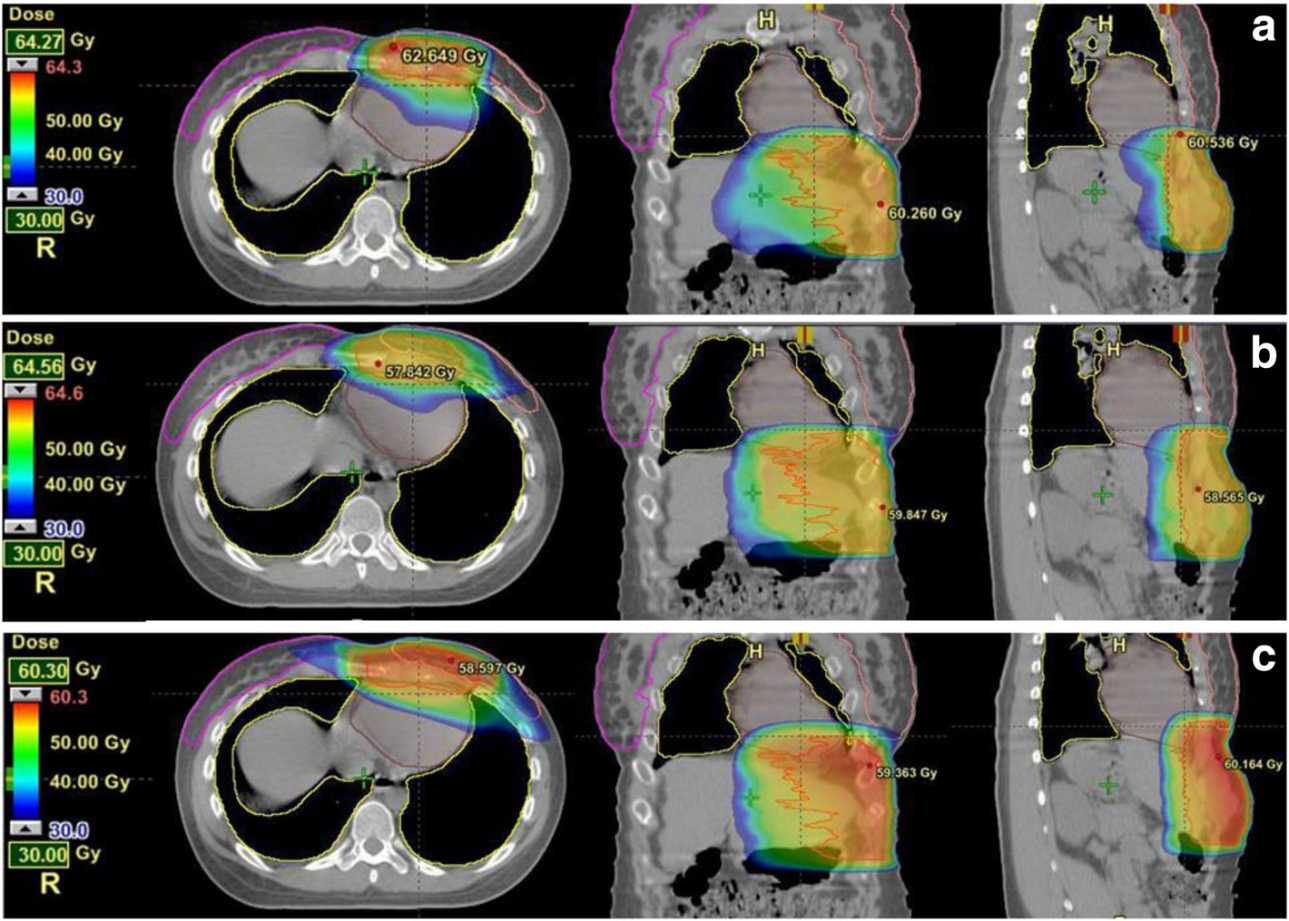
Example of dose distributions for example case for VMAT (A), IMRT (B) and 3DCRT (C)

Table 3 and Fig. 1 summarise the dosimetric results for organs at risk. The mean dose to heart was statistically similar for all three plan types. However, the mean dose to lung was significantly higher for 3DCRT (11.9Gy ± 7.9) compared to IMRT (9.4Gy ± 5.4, *p* = 0.014) and VMAT (8.9Gy ± 4.5, *p* = 0.017). There were no significant differences in the dose to lung for the IMRT plans compared to VMAT plans (*p* = 0.386). Similarly, the V20 was significantly higher for 3DCRT (21.0Gy ± 18.7) compared with IMRT (16.2Gy ± 12.4, *p* = 0.021) and VMAT (12.1 ± 7.5Gy, *p* = 0.028). For the 3 female patients, the low dose regions in the ipsilateral breast for VMAT were generally less than IMRT and 3DCRT. As shown in Fig. 1, the volume of lung and left (ipsilateral) breast irradiated at low and medium doses was lower for IMRT and VMAT compared to 3DCRT. The DVH for heart and the right (contralateral) breast were very similar. A typical plan for a female patient with a large left sided anterior chest wall desmoid tumour is shown in Fig. 2, illustrating the improved OAR sparing achieved with VMAT and IMRT compared to 3DCRT.

**Table 3:** Summary of dosimetric results for Organs At Risk

Parameter	Patients	Parameter	3DCRT	IMRT	VMAT	*P* value		
						3DCRT vs IMRT	3DCRT vs VMAT	IMRT vs VMAT
Heart	10/10	Mean (Gy)	8.3 ± 7.6	5.7 ± 3.7	6.5 ± 4.3	0.074	0.333	0.093
		V_25Gy_ (%)	8.1 ± 14.3	3.6 ± 4.0	3.9 ± 4.9	0.310	0.735	0.917
		V_30Gy_ (%)	5.6 ± 10.0	3.9 ± 5.4	2.3 ± 3.5	0.237	0.735	0.753
Lung	10/10	Mean (Gy)	11.9 ± 7.9	9.4 ± 5.4	8.9 ± 4.5	0.014	0.017	0.386
		V_20Gy_ (%)	21.0 ± 18.7	16.2 ± 12.4	12.1 ± 7.5	0.021	0.028	0.515
Right breast (contralateral breast)	3/10	Mean (Gy)	3.3 ± 0.5	2.7 ± 0.5	3.5 ± 1.6	0.285	0.593	0.285
		V_10Gy_ (%)	2.6 ± 3.0	2.7 ± 2.4	5.2 ± 7.7	1.000	1.000	0.655
		V_20Gy_ (%)	1.4 ± 2.1	0.5 ± 0.8	0.4 ± 0.7	0.180	0.655	0.655
Left breast (ipsilateral breast)	3/10	Mean (Gy)	19.7 ± 12.7	11.4 ± 6.8	11.1 ± 7.6	0.109	0.109	0.593
		V_10Gy_ (%)	44.4 ± 36.2	38.9 ± 31.6	28.8 ± 20.4	0.180	0.285	0.593
		V_20Gy_ (%)	28.9 ± 20.0	18.2 ± 13.6	15.7 ± 18.4	0.285	0.593	0.593

Mean (Gy) refers to the mean dose to organ at risk. V_25Gy_ (%) refers to the percentage of the volume receiving 25 Gy dose. P value determined using Wilcoxon matched pair rank sum test in SPSS

## Discussion

This is the first comparative planning study on the performance of 3DCRT, IMRT and VMAT plans for patients with unresectable chest wall desmoid tumours undergoing definitive RT. Our findings are consistent with a large body of literature demonstrating that VMAT achieves highly conformal dose distribution with better target volume coverage and sparing of normal tissues compared with conventional techniques (reviewed in [10]). In particular, PTV coverage was found to be similar for IMRT and VMAT, with both techniques having superior conformity index compared to 3DCRT. Similar findings have been reported in planning studies of other tumour sites including prostate [11], anal [12] head and neck [13] and CNS tumours [14].

Definitive RT is emerging as an important treatment modality for desmoid tumours. A multicentre phase II study of primary RT for inoperable desmoid tumours showed moderate dose RT as an effective treatment option with absence of local progression in 81.5 % of subjects at 3 years [9]. However due to the size and difficult location of chest wall desmoid tumours, recurrence and RT treatment sequelae can considerably impair functional outcomes and quality of life. In the current study, a dose of 56 Gy in 28 fractions was chosen based upon existing literature showing radiation doses over 56 Gy did not significantly improve local control but were associated with increased risk of complications [8].

We showed that VMAT required 60 % less MUs on the machine compared to IMRT in this study. The lower MUs is also associated with less interleaf scatter dose and reduced radiotherapy dose to normal tissues, thus minimising the risk of second radiation-induced malignancies [15] which is of particular relevance when delivering radiotherapy to young patients with desmoid tumours (age range 20–64 in our study). VMAT was found to have similar beam on time compared to IMRT and both required more beam on time compared to 3DCRT. However, the large number of fixed gantry field with IMRT approach increases the total treatment time compared with VMAT and this is likely to impact on intrafractional patient movement and clinical throughput of the radiotherapy department.

As expected, this study found that dose to the lung and ipsilateral breast was also substantially lower for IMRT and VMAT compared to 3DCRT (Fig. 2). Additionally VMAT had a trend towards lower ipsilateral breast dose compared to IMRT, although the small numbers of female patients in our study precluded an ability to assess statistical significance of this finding. Nevertheless, reduced OAR dose is important to minimise late effects given that desmoid tumour is a benign condition primarily affecting young patients. There have been varying reports on OAR sparing for IMRT versus VMAT in the literature. In prostate cancer planning studies VMAT has been shown in most studies to have better sparing of rectum, bladder and urethra compared to IMRT, whereas OAR sparing was similar or only slightly better for VMAT compared to IMRT in head and neck cancers [10]. In breast cancer planning studies in a similar anatomical area to desmoid tumour plans, VMAT has shown to be better than IMRT and 3DCRT for sparing of heart and ipsilateral lung as well as the contralateral breast [16, 17]. Future studies should compare IMRT and VMAT in a larger number of female patients to clarify whether VMAT is associated with improved sparing of the ipsilateral breast, given the risk of secondary malignancies in young women.

## Conclusion

In summary, this is the first comprehensive planning study to evaluate VMAT, IMRT and 3DCRT planning techniques for unresectable chest wall desmoid tumours. Our findings show that both VMAT and IMRT provided superior PTV coverage and improved OAR sparing compared to 3DCRT. VMAT was associated with reduced MUs with a trend towards lower ipsilateral breast dose compared to IMRT. Further prospective studies are required to assess whether VMAT would translate to clinical benefits especially reduced incidence of late effects compared to IMRT, however this is challenging due to the low incidence of desmoid tumour and the long follow-up that would be required.
